# Supplementary material for: Dual-comb optical activity spectroscopy for the analysis of vibrational optical activity induced by external magnetic field
Source: Nat Commun. 2023 Feb 16;14:883. doi: 10.1038/s41467-023-36509-w (PMC9935641; doi:10.1038/s41467-023-36509-w)
Supplement: Supplementary file 1 — Supplementary Information [file 41467_2023_36509_MOESM1_ESM.pdf]

Supplementary Information for "Dual-comb optical activity spectroscopy for the analysis of vibrational optical activity induced by external magnetic field"

Daowang Peng[1,2], Chenglin Gu[1,2], Zhong Zuo[1], Yuanfeng Di[1], Xing Zou[1], LuluTang[1], Lunhua Deng[1], Daping Luo[1], Yang Liu[1] & Wenxue Li[1,*]

[1]*State Key Laboratory of Precision Spectroscopy, East China Normal University, Shanghai 200062, China.*

[2]*These authors contributed equally to this work.*

**e-mail: wxli@phy.ecnu.edu.cn.*

Supplementary Information note 1: Performance of dual-comb optical activity spectroscopy

a. Signal-to-noise ratio and detection sensitivity of dual-comb optical activity.

We further investigate the signal-to-noise ratio (SNR) and the detection sensitivity by varying the measurement time in this section. The SNR and sensitivity are defined as shown in the **Methods** section in the manuscript. Figure S1a and S1b show that the peak SNRs of MVCD and MORD both increase linearly with the square root of the measurement time. The Allan deviations of the MVCD and MORD sensitivity are depicted in Fig. S1c and S1d, respectively. The sensitivity of DC-OAS scales as $t^{-1/2}$ (t is the measurement time) even the measurement time up to 1000 s. It should be noted that the long-term coherent averaging is an effective method to improve the detection sensitivity (SNR).

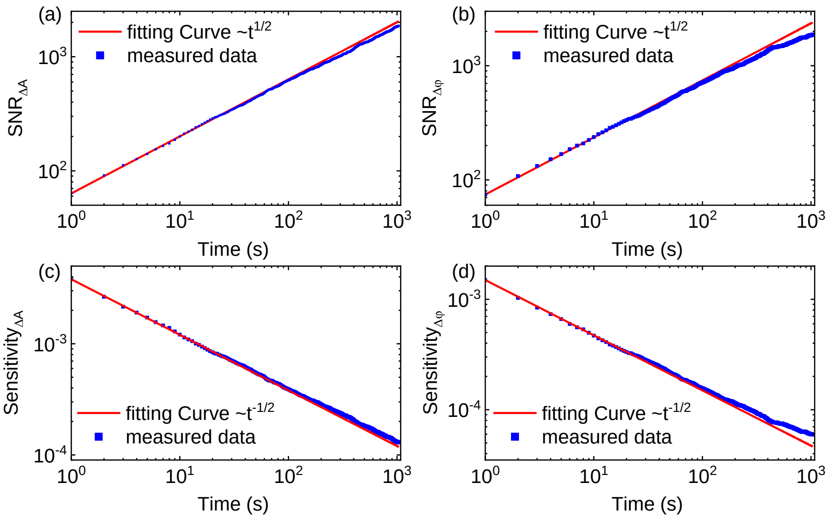

Fig. S1 a and **b** Signal-to-noise ratio (SNR) of VCD and ORD as a function of averaging time,

respectively. The solid lines are the fitting curves of the $t^{1/2}$ trends. **c** and **d** Allan deviations of the $1-\sigma$ sensitivities of VCD and ORD. The curves show the inverse square root dependency. The red solid lines are the fitting curves of the $t^{-1/2}$ trends.

b. Comparison and discussion of system performance

In this section, we compare several representative works in the table below to illustrate the advantages of our technique.

Table S1. Performance comparison with other technologies.

	Measurement time	Resolution	Sensitivity
Ref. 1	>8000 s(g)	3 GHz	~10^{-4}
Ref. 2	3500 s (g)	250 MHz	~10^{-3}
Ref. 3	Tens of minutes (l)	90 GHz	>10^{-5}
Our work	1000 s (g)	108 MHz	~10^{-4}
	5.4 s (l)[#]	72 GHz	>10^{-5}

g: gas-phase system   l: liquid-phase system

The total sampling time lasts only 5.4 s within 1-hour measurement time.

In the gas-phase OA experiments, Timothy A. Keiderling et al.[1] have investigated the magnetic VCD spectra of a large number of gas-phase paramagnetic/ferromagnetic molecules using a Fourier transform spectrometer, achieving a sensitivity of ~10^{-4} (total measurement time exceeds 8000 s) and a resolution of 3 GHz. Aleksandra Foltynowicz's group[2] attempted to apply the Fourier-transform-spectrometer-based optical frequency comb to Faraday rotation spectroscopy, obtaining a spectral resolution of 250 MHz and a sensitivity of ~10^{-3} @3500 s at the expense of long-range scanning. In contrast, DC-OAS is based on optical asynchronous sampling without any mechanical scanning, and the single spectral refresh time can be reduced to the order of microseconds. Moreover, the frequency-domain multi-heterodyne interference of dual-comb spectrometer down-converts the spectral information to the radio frequency, which suppresses the white noise[4] ($\propto 1/f$) and greatly improves the detection sensitivity. Our scheme has simultaneously measured VCD and ORD spectra with a resolution of 108 MHz and a sensitivity of ~10^{-4} @1000 s, which offers significant improvements in gas-phase OA analysis.

In the liquid-phase OA experiments, Minhaeng Cho's group provides orders of magnitude improvement in the measurement speed of weak chiroptical activity (COA) signals. They claimed that their technique, a heterodyned spectral interferometry based on the cross-polarization detection, realized the COA measurement of limonene (10^{-5} smaller than absorption) with a resolution of 90 GHz and reduced the measurement time from multiple hours to tens of minutes. In our experiment, the VCD

and ORD spectra of limonene were simultaneously achieved in the 5.4-s sampling time with 72-GHz resolution. However, limited by the low duty cycle of the low-repetition-rate system, the total measurement time lasted 1 hour. It would be ideal if the repetition rate f_r of optical frequency comb was equal to the desired resolution, and the measurement time could be reduced by more than five orders of magnitude from 1 hour to millisecond level. With the development of the high-repetition-rate sources, such as microcavity[5] and quantum cascade laser[6], our technique has unparalleled advantages in the rapid measurement of optical activity.

Supplementary Information note 2: Simulation of the Zeeman effect

a. NO_2

The Zeeman behaviour of NO_2 has been explained by CD Hause[7], TJ Sears[8] and JT Hougen[9] et al. In the external magnetic field, the total Hamiltonian of NO2 consists of the vibration-rotation Hamiltonian, the spin-rotation interaction Hamiltonian and the Zeeman Hamiltonian,

$$H_{tot} = H_{\mathrm{vr}} + H_{\mathrm{sr}} + H_{\mathrm{z}}.$$

The matrix element of the spin-rotation interaction Hamiltonian is given by[10]

$$\langle N, K_a, S, J | H_{\mathrm{sr}} | N, K_a, S, J \rangle = \gamma_{sr} \frac{J(J+1) - N(N+1) - S(S+1)}{2}$$

The parameter is γ_{sr} defined as $\gamma_{sr} = \left[\bar{\varepsilon}_{bb} + \frac{(\varepsilon_{aa} - \bar{\varepsilon}_{bb}) {K_a}^2}{N(N+1)}\right]$, where $\bar{\varepsilon}_{bb} = \frac{\varepsilon_{bb} + \varepsilon_{cc}}{2}$; ε_{aa}, ε_{bb} and ε_{cc} are the spin-rotation interaction constants. The Zeeman Hamiltonian H_{z} of NO2 can be written as[7]

$$H_Z = -\boldsymbol{\mu} \cdot \mathbf{H} = (g_S \mu_B / \hbar) \mathbf{S} \cdot \mathbf{H}$$

where $g_S = +2.00232$ is the electron spin gyromagnetic ratio and $\mu_B = +0.046686\ \mathrm{cm}^{-1}\ \mathrm{kG}^{-1}$ is the Bohr magneton. The Hamiltonian matrix has the form[9]

$$\begin{bmatrix} E_{\mathrm{vr}} - \frac{1}{2}\gamma_{sr}\left(M_J + \frac{1}{2}\right) - \frac{1}{2} g_{\mathrm{B}} H, & +\frac{1}{2}\gamma_{sr}\left[\left(N + \frac{1}{2}\right)^2 - {M_J}^2\right]^{1/2} \\ +\frac{1}{2}\gamma_{sr}\left[\left(N + \frac{1}{2}\right)^2 - {M_J}^2\right]^{1/2}, & E_{\mathrm{vr}} + \frac{1}{2}\gamma_{sr}\left(M_J - \frac{1}{2}\right) + \frac{1}{2} g_{\mathrm{B}} H \end{bmatrix}.$$

This matrix must be replaced by a matrix containing only the lower right (upper left) diagonal element when $M_J = \pm\left(N + \frac{1}{2}\right)$. Then, molecular energy levels in the magnetic field are given by the eigenvalues of the matrix,

$$\mathrm{E}(\mathrm{N}, M_J, \pm 1/2) = E_{\mathrm{vr}} - \frac{1}{4}\gamma_{sr} \pm \frac{1}{2}\left[{\gamma_{sr}}^2 \left(N + \frac{1}{2}\right)^2 + 2\gamma_{sr} M_J g_S \mu_{\mathrm{B}} H + {g_s}^2 \mu_{\mathrm{B}}^2 H^2\right]^{\frac{1}{2}},$$

for $|M_J| \leq \left(N - \frac{1}{2}\right)$, and

$$E = E_{\mathrm{vr}} + \frac{1}{2}\gamma_{sr}N \pm \frac{1}{2}g_s\mu_{\mathrm{B}}H$$

for $M_J = \pm\left(N + \frac{1}{2}\right)$. Through the direction cosines in Table S1[11], the transition probability for an electric dipole transition from a state $|n\rangle$ to a state $|m\rangle$ is given by

$$|\langle n|M_A|m\rangle|^2 = \left|\sum_{\alpha} M_\alpha(\Phi_{A\alpha})_{n,m}\right|^2$$

Here, $A = X, Y, \mathrm{or}\, Z$ and $\alpha = x, y, \mathrm{or}\, z$.

Table S2. Values of the elements of all direction-cosine matrices for the prolate-symmetric-top molecule, NO_2.

Matrix-element factor	$\Delta J = +1$	$\Delta J = 0$	$\Delta J = -1$
$(\Phi_{A\alpha})_{J,J'}$	$[4(J+1)\sqrt{(2J+1)(2J+3)}]^{-1}$	$[4J(J+1)]^{-1}$	$\left[4J\sqrt{4J^2-1}\right]^{-1}$
$(\Phi_{Az})_{J,K_a;J',K_a}$	$2\sqrt{(J+K_a+1)(J-K_a+1)}$	$2K_a$	$-2\sqrt{J^2-{K_a}^2}$
$(\Phi_{Ay})_{J,K_a;J',K_a\pm1} = (\Phi_{Ax})_{J,K_a;J',K_a\pm1}$	$\mp\sqrt{(J\pm K_a+1)(J\pm K_a+2)}$	$\sqrt{(J\mp K_a)(J\pm K_a+1)}$	$\mp\sqrt{(J\mp K_a)(J\mp K_a-1)}$
$(\Phi_{Z\alpha})_{J,M_J;J',M_J}$	$2\sqrt{(J+M_J+1)(J-M_J+1)}$	$2M_J$	$-2\sqrt{J^2-{M_J}^2}$
$(\Phi_{Y\alpha})_{J,M_J;J',M_J\pm1} = \pm i(\Phi_{X\alpha})_{J,M_J;J',M_J\pm1}$	$\mp\sqrt{(J\pm M_J+1)(J\pm M_J+2)}$	$\sqrt{(J\mp M_J)(J\pm M_J+1)}$	$\mp\sqrt{(J\mp M_J)(J\mp M_J-1)}$

b. NO

For the diatomic molecule of nitric oxide, the additional energy due to the magnetic field can be expressed as

$$\Delta E = -M_J g_J \mu_{\mathrm{B}} B$$

where M_J are the magnetic quantum numbers, g_J is the Lande g-factor, μ_{B} is the Bohr magneton, and B is the magnetic field strength. The ro-vibrational transitions between the Zeeman sublevels of $v'J'M'_{J'}$ and $v''J''M''_{J''}$ are given by

$$v_{v'J'M'_{J'}\leftarrow v''J''M''_{J''}} = v_0 + \left(M'_{J'}g^{v'}_{J'} - M''_{J''}g^{v''}_{J''}\right)\frac{\mu_{\mathrm{B}}B}{hc}$$

where vJM_J are vibrational quantum numbers, rotational quantum numbers and magnetic quantum numbers, and the primes and double primes refer to upper and lower states, respectively. h is the Planck constant, c is the speed of light, and v_0 is the transition frequency without the external field. The Lande g-factor g_J of NO is given by Hund's coupling case (a–b)[12,13],

$$g_J = \frac{3/2 \pm [2(J-1/2)(J+3/2) - 3Y/2 + 3]/X}{J(J+1)}$$

where $X = \sqrt{4(J-1/2)(J+3/2)+(Y-2)^2}$ and $Y = A_v/B_v$, A_v and B_v are the spin–orbit coupling and rotational constants of the vibrational quantum number v, respectively; the upper and lower signs represent the $X^2\Pi_{1/2}$ and $X^2\Pi_{3/2}$ fine-structure components, respectively. For the overtone $(2 \leftarrow 0)$ band of NO, $A_0 = 123.13361\ \mathrm{cm}^{-1}$, $B_0 = 1.6961483\ \mathrm{cm}^{-1}$ and $A_2 = 122.63486\ \mathrm{cm}^{-1}$, $B_0 = 1.6609632\ \mathrm{cm}^{-1}$ [14,15]. The centre frequency of the Zeeman transitions $\sigma^{\pm}$ $(\Delta M_J = \pm 1)$ is described by

$$\nu_{\sigma^{\pm}} = \nu_0 + \left[\left(M''_{J''} \pm 1\right)g^{v'}_{J'} - M''_{J''}g^{v''}_{J''}\right]\frac{\mu_B B}{hc}.$$

As shown in Table S2, the line intensities of the Zeeman subtransitions, $S_{M'M''} = \bar{S}_{M'M''} \cdot S_{\Omega J'J''}$, can be calculated by the corresponding 3J symbol[16]

$$\bar{S}_{M'M''} = \begin{pmatrix} J' & 1 & J'' \\ -M' & \Delta M & M'' \end{pmatrix}^2 \text{ and } \sum_{M'M''}\bar{S}_{M'M''} = 1.$$

The integrated line intensity $S_{\Omega J'J''}$ can be obtained in the HITRAN database, as well as the zero-field parameters, such as the transition frequency and the quantum numbers of the upper levels and lower levels.

Table S3. Table of relative strength, $\bar{S}_{M'M''}$, for the Zeeman transitions.

ΔJ	$\Delta M_J = +1$	$\Delta M_J = -1$
+1	$\frac{(J+M_J+1)(J+M_J+2)}{2(J+1)(2J+1)(2J+3)}$	$\frac{(J-M_J+1)(J-M_J+2)}{2(J+1)(2J+1)(2J+3)}$
0	$\frac{(J-M_J)(J+M_J+1)}{2J(J+1)(2J+1)}$	$\frac{(J+M_J)(J-M_J+1)}{2J(J+1)(2J+1)}$
-1	$\frac{(J-M_J)(J-M_J-1)}{2J(2J+1)(2J-1)}$	$\frac{(J+M_J)(J+M_J-1)}{2J(2J+1)(2J-1)}$

Supplementary Information note 3: Calculation of the MVCD and MORD spectra

The transition frequency $\nu_{\pm}$ and the line strength $S_{\pm}$ of the Zeeman components $(\sigma^{\pm})$ under a magnetic field are given by the above analysis. The transmission spectra are described as[17]

$$T_{\pm}(\nu) = \exp\left[-\phi(\nu_{\pm})S_{\pm}\frac{P}{P_0}N_0\frac{T_0}{T}L\right]$$

where $\phi(\nu_{\pm})$ is the Voigt-shape function, N_0 is the number density at $T_0 = 273.15K$ and $P_0 = 1000$ mbar, P and T are the temperature and pressure under the experimental conditions. In order to reduce the computation time in full-vibrational-band simulation, the absorption profile is expressed by the pseudo-Voigt function[18],

$$F_{pV} = (1-\eta)F_G(\nu;\gamma_G) + \eta F_L(\nu;\gamma_L),$$

$$F_G(\nu;\gamma_G) = 1/\pi^{1/2}\gamma_G e^{-\nu^2/\gamma_G^2} \text{ and } F_L(\nu;\gamma_L) = 1/(\pi\gamma_L)(1+\nu^2/\gamma_L^2)^{-1},$$

where ν is the center frequency of the absorption transition, $F_G(\nu;\gamma_G)$ and $F_L(\nu;\gamma_L)$

are the Gaussian and Lorentzian functions, η is the mixing-parameter of the two functions,

$$\eta = 1.36603\gamma_L/\gamma - 0.47719(\gamma_L/\gamma)^2 + 0.11116(\gamma_L/\gamma)^3,$$

$$\gamma = (\gamma_G^5 + 2.69299\gamma_G^4\gamma_L + 2.42843\gamma_G^3\gamma_L^2 + 4.47163\gamma_G^2\gamma_L^3 + 0.07842\gamma_G^1\gamma_L^4 + \gamma_L^5)^{1/5}.$$

The profile parameters and the measurement parameters used in the molecular simulations are shown in the following Table S4.

Table S4. Parameters used in the simulation.

	γ_D/cm^{-1}	γ_L/cm^{-1}	$P/mbar$	T/K	L/cm
NO_2 for Faraday effect	$9.11 * 10^{-7}v_0$*	0.005	8	298	7.6
NO_2 for Voigt effect	$9.11 * 10^{-7}v_0$*	0.0023	1.7	298	30
NO	$11.28 * 10^{-7}v_0$*	0.012	65	298	7.6

*v_0 is the wavenumber of the transition line.

And due to the mode-resolved characteristics of our dual-comb spectrometer, no instrumental lineshape was taken into account[19]. The MVCD (ΔA) is obtained by calculating the difference in absorbance between $\sigma^{\pm}$,

$$\Delta A = -\lg T_+(v) + \lg T_-(v) = -\lg \frac{T_+(v)}{T_-(v)}.$$

According to the Kramers-Kronig relationship[20], the phase spectra of the Zeeman transitions could be calculated by

$$\varphi_{\pm}(v) = \frac{2}{\pi}\int_0^{+\infty} \frac{v' \ln T_{\pm}(v)}{v'^2 - v^2} dv'.$$

And the MORD ($\Delta\varphi$) is expressed by

$$\Delta\varphi = \frac{\varphi_+(v) - \varphi_-(v)}{2}.$$

The calculations of magnetic linear dichroism and magnetic birefringence in a transverse magnetic field can be performed easily by replacing σ^+/σ^- with σ/π.

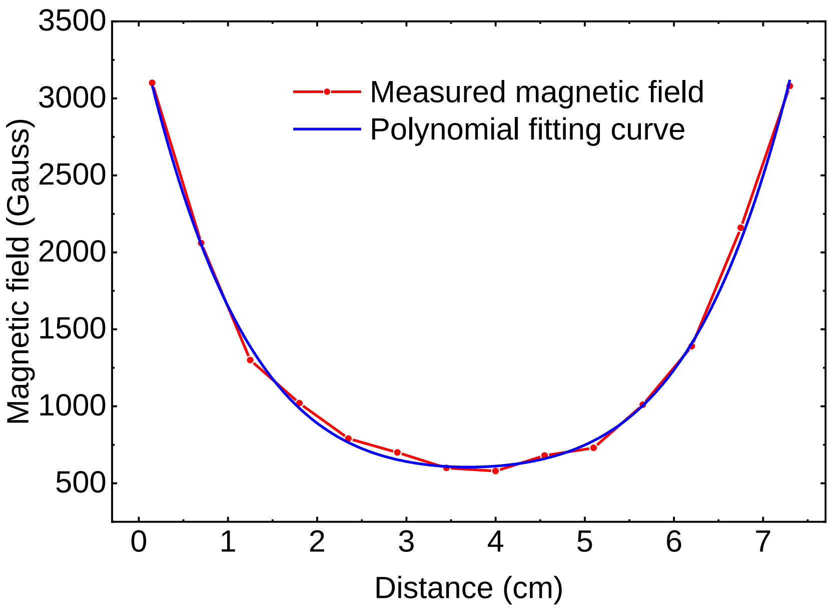

Fig. S2 Measured magnetic field (red curve) and polynomial fitting curve (blue curve).

In the simulation, the inhomogeneous distribution of the magnetic field should be considered. As shown in Fig. S2, the distribution of the longitudinal magnetic field strength along the cell is measured by using a Gaussmeter. A polynomial fit was applied to characterize the strength distribution of our magnetic field along the propagation direction of the incident beams. The fitting curve can be expressed as:

$$B(d) = 9.91d^4 - 147.54d^3 + 892.68d^2 - 2553.13d + 3447.66$$

Based on the polynomial fitting function of the magnetic-field distribution and the molecular models of NO_2 and NO, calculations of the MOA spectra are carried out to explain the experimental results.

Reference

1. Tam, C. N. & Keiderling T. A. Direct measurement of the rotational g-value in the ground vibrational state of acetylene by magnetic vibrational circular dichroism. *Chem. Phys. Lett.* **243**, 55-58 (1995).
2. Johansson, A. C., Westberg, J., Wysocki, G. & Foltynowicz, A. Optical frequency comb Faraday rotation spectroscopy. *Appl. Phys. B* **124**, 1-8 (2018).
3. Rhee, H. et al. Femtosecond characterization of vibrational optical activity of chiral molecules. *Nature* **458**, 310-313 (2009).
4. Newbury, N. R., Coddington, I. & Swann, W. Sensitivity of coherent dual-comb spectroscopy. *Opt. Express* **18**, 7929-7945, (2010).
5. Yu, M., Okawachi, Y., Griffith, A. G., Picqué, N., Lipson, M. & Gaeta, A. L. Silicon-chip-based mid-infrared dual-comb spectroscopy. *Nat. Comm.* **9**, 1-6 (2018).
6. Hillbrand, J., Andrews, A. M., Detz, H., Strasser, G. & Schwarz, B. Coherent injection locking of quantum cascade laser frequency combs. *Nat. Photon.* **13**, 101-104 (2019).
7. Olman M. D. & Hause C. D. Analysis of the high-resolution zeeman spectra of nitrogen dioxide in the

near infrared. *J. Chem. Phys.* **49**, 4575-4583 (1968).

8. Brown J. M. & Sears T. J. A determination of Zeeman parameters for NO_2 in its ground state. *Mol. Phys.* **34**, 1595-1610 (1977).
9. Hougen J. T. The assignment of molecular infrared spectra from a laser magnetic resonance spectrometer. *J. Mol. Spectrosc.* **54**, 447-71 (1975).
10. Tada K., Hirata M. & Kasahara S., Hyperfine interaction constants of $^{14}NO_2$ in 14 500–16 800 cm^{-1} energy region. *J. Chem. Phys.* **147**, 164304 (2017).
11. Cross P. C., Hainer R. M. & King G. W. The asymmetric rotor II. Calculation of dipole intensities and line classification. *J. Chem. Phys.* **12**, 210-243 (1944).
12. Radford H. E., Microwave Zeeman effect of free hydroxyl radicals. *Phys. Rev.* **122**, 114 (1961).
13. Herrmann W., Rohrbeck W. & Urban W. Line shape analysis for Zeeman modulation spectroscopy. *Appl. Phys.* **22**, 71-75 (1980).
14. Brown J. M., Kaise M., Kerr C. M. L. & Milton D. J. A determination of fundamental Zeeman parameters for the OH radical. *Mol. Phys.* **36**, 553-582 (1978).
15. Pine A. S., Johns J. W. C. & Robiette A. G. Λ-Doubling in the v= 2← 0 overtone band in the infrared spectrum of NO. *J. Mol. Spectrosc.* **74**, 52-69 (1979).
16. Borkov Y. G., Klimachev Y. M. & Sulakshina O. N. Dependence of Zeeman splitting of spectral lines on the magnetic field magnitude for NO molecule. *Atmos. Ocean. Opt.* **29**, 103-118 (2016).
17. Li J., Yu B., Zhao W. & Chen W. A review of signal enhancement and noise reduction techniques for tunable diode laser absorption spectroscopy. *Appl. Spectrosc. Rev.* **49**, 666-691 (2014).
18. Ida, T., Ando, M. & Toraya, H.. Extended pseudo-Voigt function for approximating the Voigt profile. *J. Appl. Crystallogr.* **33**, 1311-1316 (2000).
19. Picqué, N. & Hänsch, T. W. Frequency comb spectroscopy. *Nat. Photon.* **13**, 146-157 (2019).
20. Szabo, Z., Park, G. H., Hedge, R. & Li, E. P. A unique extraction of metamaterial parameters based on Kramers–Kronig relationship. *IEEE T. Microw. Theory* **58**, 2646-2653 (2010).
